# Evidence of porcine epidemic diarrhea virus (PEDV) shedding in semen from infected specific pathogen-free boars

**DOI:** 10.1186/s13567-018-0505-2

**Published:** 2018-01-24

**Authors:** Sarah Gallien, Angélique Moro, Gérald Lediguerher, Virginie Catinot, Frédéric Paboeuf, Lionel Bigault, Mustapha Berri, Phillip C. Gauger, Nathalie Pozzi, Edith Authié, Nicolas Rose, Béatrice Grasland

**Affiliations:** 1 Anses, Laboratory of Ploufragan/Plouzané, BP53, 22440 Ploufragan, France; 2 Université Bretagne Loire, 35000 Rennes, France; 3 Institut National de Recherche Agronomique (INRA), UMR 1282, Université François Rabelais, 37380 Nouzilly, France; 4 Laboratoire National de Contrôle des Reproducteurs (LNCR), 94700 Maisons-Alfort, France; 5 Veterinary Diagnostic & Production Animal Medicine, Veterinary Diagnostic Laboratory, College of Veterinary Medicine, Iowa State University, Ames, IA 50011 USA

## Abstract

In 2013, PED emerged for the first time in the United States (US). The porcine epidemic diarrhea virus (PEDV) spread quickly throughout North America. Infection with PEDV causes watery diarrhea and up to 100% mortality in piglets, particularly for highly pathogenic non-InDel strains circulating in the US. PEDV is mainly transmitted by the fecal–oral route. Transmission via the venereal route has been suspected but not previously investigated. The aim of the study was to determine if PEDV could be detected in semen from infected specific pathogen-free (SPF) boars inoculated with a PEDV US non-InDel strain suggesting venereal transmission may occur. Two boars orally inoculated with PEDV showed clinical signs and virus shedding in feces. Transient presence of the PEDV genome was detected by RT-qPCR in the seminal (5.06 × 10^2^ to 2.44 × 10^3^ genomic copies/mL) and sperm-rich fraction of semen (5.64 × 10^2^ to 3.40 × 10^4^ genomic copies/mL) and a longer duration of viral shedding was observed in the sperm-rich fraction. The evidence of PEDV shedding in semen raises new questions in term of disease spread within the pig population with the use of potentially contaminated semen.

## Introduction

Porcine epidemic diarrhea (PED) emerged for the first time in Europe during the 1970s [1]. The virus responsible for this disease is an *alphacoronavirus* known as porcine epidemic diarrhea virus (PEDV). The PEDV has a single-stranded, positive-sense RNA genome. An infection with PEDV in pigs leads to severe liquid diarrhea, vomiting and dehydration. In suckling piglets population, PEDV causes high mortality [2]. In older animals, morbidity may approach 100%, but mortality remains low between 1 and 3% [3]. Economic losses due to this virus are significant [2, 4–7]. Currently, PEDV is present on the Asian, American and European continents. In 2013, 40 years after the first cases of PED in Europe, the disease emerged in the United States (US), in the Midwestern state of Iowa, and ultimately affected the swine population of the entire country; until this date, the US had been free from PED [8]. The virus spread rapidly causing a major impact on pig production including mortality of 10% of the total US swine production or approximately 7 million piglets in less than 1 year [9]. Two genetically strains of PEDV have been identified in the USA, namely, the “non-InDel” strain, close to the aforementioned Asian strains, and the “InDel” strain, showing insertion-deletion in the S1 part of the S gene. Currently, PEDV is endemic in the US with periodic waves of infection occurring mostly during winter. The virus also spread to others countries in North and South America, such as Canada and Mexico [2, 10, 11]. In Europe, PED cases associated with the InDel strains have been detected since 2014 [10, 12–14].

Different PEDV transmission routes have been identified. The major mode of transmission is the fecal–oral route through direct or indirect contact with infected pigs or contaminated feces [2, 15]. Transmission of PEDV also occurs through contact with contaminated equipment, contaminated vehicles used for animal transport, or farm employees [2, 16, 17]. Experimental studies have shown that airborne samples containing PEDV can be infectious for pigs so PEDV could be spread through aerosols [18]. In addition, PEDV has been transmitted vertically through contaminated milk from the sow to its piglets [5]. PEDV transmission through semen has also been a concern due to a viremia that occurs during the acute phase of infection during which the virus may be recovered in blood and sexual organs [10]. Contamination of semen by PEDV during processing may also occur. A recent study demonstrated PEDV nucleic acid detection in infected boars’ semen; however, the authors were not able to rule out external contamination by fecal material during collection and/or preparation of the semen [19]. The PEDV genome has also been detected in the semen of healthy boars from three different farms in China. The quantity of detected PEDV genome ranged from 10^1.46^ to 10^3.65^ genome copies/mL of semen [20]. It is known that some viruses such as porcine reproductive and respiratory syndrome virus (PRRSV), porcine parvovirus (PPV), African swine fever virus (ASFV), classical swine fever virus (CSFV), foot and mouth disease virus (FMDV), Japanese B encephalitis virus (JBEV) and porcine circovirus type 2 (PCV2) are shed in semen [21]. However, PEDV detection in and transmission via semen has not been previously studied in experimentally infected boars. Contamination of semen by different porcine viruses via contaminated feces or aerosols could be possible [22]. In France, non-InDel strains of PED are classified in the first category of regulated animal health hazards (Order Number: AGRG1410808A). In order to maintain the PEDV free status for non-InDel strains in France and in Europe, importing boars and boar semen from countries where non-InDel strains are endemic is strictly controlled. Therefore, it is important to know if PEDV can be shed in semen from infected boars in order to determine appropriate biosecurity measures necessary to prevent transmission of the virus. The objective of this study was to determine if PEDV can be shed in semen from specific pathogen-free (SPF) boars infected by a US non-InDel strain of PEDV.

## Materials and methods

### Animals and experimental design

The experiment was carried out in the air-filtered level 3 biosecurity facilities of the French Agency for Food, Environmental and Occupational Health & Safety (ANSES) in accordance with the European and French regulations on animal welfare. The protocol for this experiment was approved by the Ethics Committee registered under number #16 by the French Ministry of Research (Referral No.16-083). Four Large White specific pathogen free (SPF) boars were included in the experimental trial, with two inoculated boars housed in the same room (Room 1: boars F1 and F2). The room contained two separate pens with one boar in each. The two remaining boars C1 and C2 (two and a half years-old) used as negative controls were housed in the ANSES SPF herd. The two boars in room 1 F1and F2 (two and a half years-old and one and a half year-old respectively) were orally inoculated in the morning of day 0 with a 5 mL homogenate of a non-InDel strain, PEDV/USA/2014/IOWA (GenBank Number: MF373643). The inoculum was prepared by homogenizing the jejunums (20% w/v) collected from PEDV infected pigs from a herd in the state of Iowa (USA) in 2014. The homogenate was centrifuged at 10 000 × *g* for 10 min at 4 °C and the supernatant filtered through a 0.45 µm filter. The complete genome of the PEDV/USA/2014/IOWA strain was sequenced by next-generation sequencing (NGS) according the protocol already described [23] and no additional RNA viral sequences were identified. The absence of PCV2 and PRSSV in the inoculum was assessed by absence of seroconversion against PCV2 and PRSSV after inoculation. The inoculum contained 10^8^ genomic copies/mL based on a quantitative real-time reverse transcriptase PCR (RT-qPCR) assay. The experiment lasted for 52 days post-inoculation (dpi). Clinical signs (lethargy, outward appearance, behavior, breathing, and diarrhea) were recorded daily. A scoring system was used for the consistency of the feces (0: absence of feces, 1: normal, 2: semi-liquid without a formed consistency, and 3: liquid/watery contents). PEDV shedding was assessed in fecal samples collected daily from the inoculated boars the first week after infection, and then three times a week until 52 dpi, using a RT-qPCR. Semen was collected before inoculation and every day during the first week post-infection and twice weekly thereafter. Serum was collected prior to inoculation and at 29 dpi in order to evaluate seroconversion. All samples were stored at –80 °C. At 52 dpi, on necropsy, the following tissue samples were collected and stored at –80 °C in RNA later tissue storage reagent (Sigma, Saint Louis, USA): duodenum, jejunum, ileum, colon, spleen, liver, mesenteric and inguinal lymph nodes, Peyer’s patches (jejunum and ileum), psoas muscle, lungs, vas deferens (right and left), testicles (right and left: apical pole, distal pole, median axis), epididymis (right and left: head, body, tail), prostate, Cowper’s glands (right and left), seminal vesicles (right and left: apical pole and distal pole) and spermatic cords (right and left). Macroscopic lesions were also evaluated on necropsy. The boars C1 and C2 were not necropsied at the end of the study.

### Semen

In order to prevent any contamination of the semen from feces or aerosols, the belly and the sheath of the boars were cleaned with cleaning wipes prior to sample collection. A swab of the prepuce was also sampled before each collection to rule out the possibility of external contamination of semen. The semen samples were collected, manually, using a collection dummy without sexual stimulation. The gelatin plug was also collected at the end of the semen ejaculate. After each collection, the fresh semen was centrifuged at 8000 × *g* for 20 min at 4 °C [24]. Through this centrifugation step, the sperm-rich fraction and the seminal fraction were separated. The two fractions were stored separately at **–**80 °C.

### Feces and tissue homogenization

Fecal homogenates were prepared with 1 mL or 1 g of feces homogenized with 9 mL of Dulbecco’s phosphate-buffered saline (Sigma-Aldrich, Saint Louis, MO, USA). The homogenates were then centrifuged at 15 000 × *g* for 10 min at 4 °C, and the supernatants were stored at **–**80 °C.

A homogenate of each tissue was prepared at 20% (w/v) in PBS (phosphate buffer saline) using a bead mill (Retsch, Haan, Germany). Subsequently, the suspensions were centrifuged at 10 000 ×* g* for 10 min at 4 °C and the supernatants were stored at **–**80 °C.

### Quantification of PEDV genomes

RNAs were extracted from the feces, tissue homogenate and from the prepuce swab supernatants using a Qiagen RNeasy Mini Kit (Qiagen, Hilden, Germany), according to the manufacturer’s instructions. RNAs were also extracted from the sperm-rich fraction, the seminal fractions and from the gelatin plugs using TRIzol^®^ assay (Thermo Fisher Scientific, Waltham, MA, USA). A total of 750 µL of TRIzol were added to 250 µL of sample and homogenized by hand. Next, 200 µL of chloroform were added and mixed by hand during 15 s and incubated at room temperature for 15 min. The tubes were centrifuged at 12 000 × *g* for 15 min at 4 °C. After centrifugation, the upper aqueous layer containing RNA was transferred to other tubes and 300 µL of TRIzol was added. The tubes were homogenized by hand for 15 s and were incubated at room temperature for 5 min. Then, 60 µL of chloroform were added and mixed by hand for 15 s. A new incubation step at room temperature for 15 min and a centrifugation at 12 000 × *g* for 15 min at 4 °C were conducted. The upper aqueous layers were transferred to new tubes and 150 µL of isopropanol and 1 µL of glycogen were added. The tubes were incubated at **–** 20 °C for 15 min and then centrifuged at 12 000 ×* g* for 10 min at 4 °C. The supernatant was discarded and 1 mL of 75% ethanol was added twice and centrifuged at 7500 ×* g* for 5 min at 4 °C. This step was repeated twice. After the centrifugation step, the ethanol was discarded and the tubes were air-dried to remove any traces of ethanol. Then, the RNA pellet was suspended in 50 µL of nuclease free water and 5 µL of extracted RNA was used as template for PEDV RT-qPCR. RNA extraction controls were included between every five samples to check for any PEDV contamination by replacing the sample with RNAse-free water.

The number of PEDV genomic copies was assessed with a SYBR Green real-time PCR using a Power SYBR^®^ Green RNA-to-Ct™ 1-step kit on a 7500 real-time PCR system (Thermo Fisher Scientific, Waltham, MA, USA). The RT-qPCR conditions used were a holding stage of two steps, which included a first step at 48 °C for 30 min, and a second at 95 °C for 10 min. The cycling stage included 40 repeated cycles of two steps, a first step of 15 s at 95 °C and a second step of 1 min at 60 °C. The melt curve stage was composed of four steps, a first step of 1 min at 95 °C, a second step of 1 min at 60 °C, a third step with a gradual increase in temperature with 0.35 °C for 0.3 s to obtain a temperature of 95 °C and a fourth step of 15 s at 60 °C. The primers used for the PCR were designed from the conserved regions of the PEDV nucleocapsid gene for universal detection of the strain used for inoculation (forward, 5′-CGCAAAGACTGAACCCACTAA-3′; reverse, 5′-TTGCCTCTGTTGTTACTTGGAGAT-3′) [25]. The copy numbers were quantified using a range from 10^1^ to 10^8^ copies/5 µL.

For each PCR run, a positive control containing PEDV RNA extract from a PEDV cell culture supernatant was included. Two negative controls were also included in the plate where the RNA template was replaced by RNAse-free water. One negative control was placed close to the positive control and the second at the end of the plate. All the samples were processed in duplicate. Prior to performing the PCR reactions, RNA extracted with the RNeasy Mini Kit was diluted 1:10 to avoid any PCR inhibition.

### PEDV serology

Serum samples were tested for PEDV antibodies using a commercial ELISA test, ID Screen^®^ PEDV Indirect (ID Vet, Grabels, France). The ELISA test is validated if the mean value of the positive control optical density (OD) is greater than 0.350 and if the ratio of the mean values of the positive and negative controls is greater than 3. A sample-to-positive (S/P) ratio was calculated. Samples with an S/P ratio equal to or greater than 60% were considered positive for PEDV antibodies.

## Results

### Boar infection

The boars inoculated with the PEDV strain demonstrated clinical signs with diarrhea observed during two periods: from 2 to 5 dpi and then again from 25 to 28 dpi. Vomiting was also observed in the infected boars at 2 dpi and again at 11 dpi. Both inoculated boars demonstrated a reduction in feed intake during the first 9 dpi. Boar F1, aged of two and a half years old, lost 8 kg and boar F2, aged of one and a half years old, gained only 21 kg during the experiment. None of the two control boars demonstrated clinical signs during the trial. All the boars were seronegative at **–**7 dpi. The two inoculated boars were positive for PEDV antibodies at 29 dpi. The two control boars remained seronegative at 31 dpi (Table 1).

**Table 1:** **Sample-to-positive (S/P) ratios (%) detected in the sera collected from SPF boars before and after inoculation**

Boar	Status	Days post-inoculation	
		**−**7 (%)	30 ± 1 (%)
F1	PEDV non-InDel strain inoculated	1	*60*
F2	PEDV non-InDel strain inoculated	1	*80*
C1	Control	0	0
C2	Control	0	1

Results in italic characters are positive.

### PEDV fecal shedding in boars

**Figure 1:**
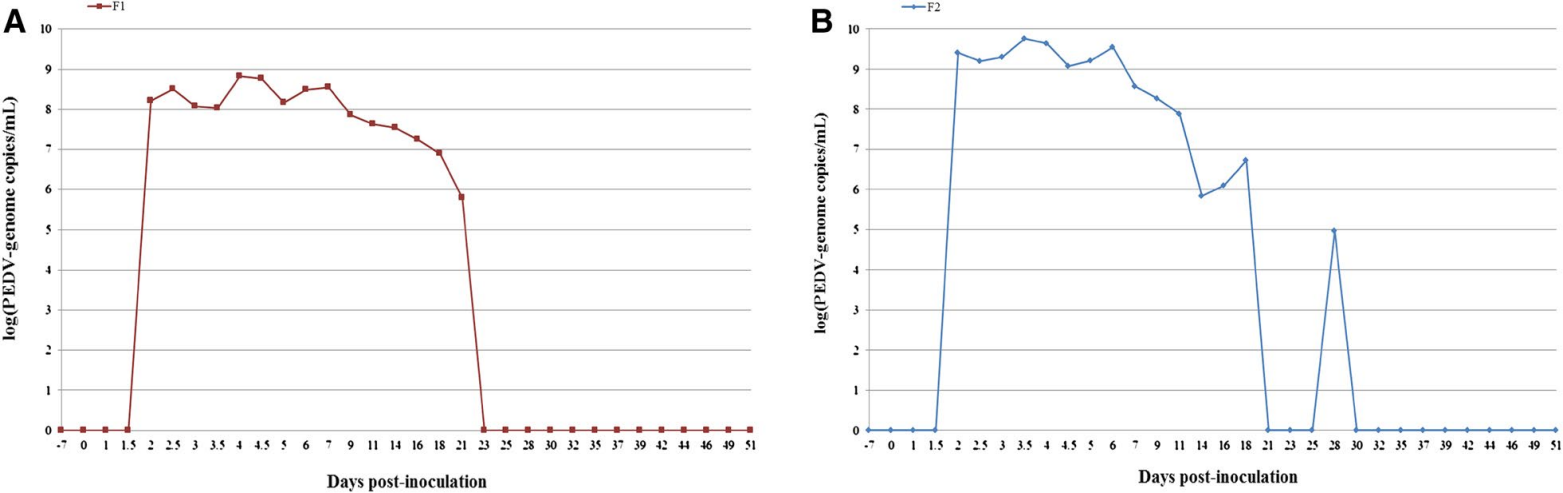
**PEDV genome loads (number of genome copies/mL) detected in feces collected from SPF boars before and after inoculation for boar F1 in A and boar F2 in B.** Individual virological data: PEDV viral genome load in feces [log(number of genome copies/mL)] in the two inoculated SPF boars (F1 and F2).

Prior to inoculation, all boars were RT-qPCR negative for PEDV in feces. The two control boars remained PEDV negative in feces throughout the trial. PEDV nucleic acid was detected in feces starting at 2 dpi with 1.62 × 10^8^ genomic copies/g and 2.53 × 10^9^ genomic copies/g for boars F1 and F2 respectively. Maximum shedding was detected between 3 and 4 dpi at 6.76 × 10^8^ genomic copies/g for boar F1 at 4 dpi, and 5.73 × 10^9^ genomic copies/g for boar F2 at 3.5 dpi. Continuous PEDV shedding was detected in boars F1 and F2 for 19 and 16 days, respectively. All feces analyzed by RT-qPCR remained under the limit of detection after 21 dpi for boar F1 and after 30 dpi for boar F2 (Figure 1).

### PEDV detection in semen and in gelatin plug

**Figure 2:**
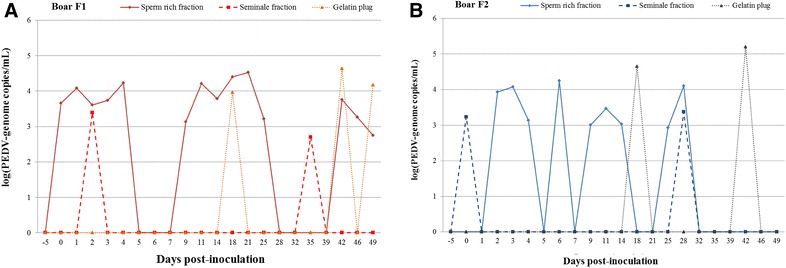
**PEDV genome loads (number of genome copies/mL) detected in semen and gelatin plugs from SPF boars before and after inoculation (A Boar F1; B Boar F2).** Individual virological data: PEDV viral genome load in sperm rich fraction, in seminal fraction and in gelatin plug [log(number of genome copies/mL)] in the two inoculated SPF boars (F1 and F2).

No PEDV RNA was detected in the semen fractions or in the gelatin plug collected 5 days before inoculation. The semen fractions and the gelatin plug collected from the control boars at 28 and 49 dpi were negative for PEDV RNA. Semen of the two PEDV inoculated boars contained PEDV RNA in both fractions. PEDV RNA was also detected in the gelatin plug of the two boars. PEDV RNA was detected for the first time in boars F1 and F2 in the sperm-rich fraction at 0.5 and 2 dpi, respectively, and in the seminal fraction at 2 and 0.5 dpi, respectively. PEDV RNA was detected in the gelatin plug from both inoculated boars at 18 dpi (Figure 2).

The detection of PEDV RNA in the seminal fraction was transient: the PEDV RNA was detected only at 2 dpi (2.44 × 10^3^ PEDV genomic copies/mL) and at 35 dpi (5.06 × 10^2^ PEDV genomic copies/mL) for boar F1, and in the afternoon the day after inoculation (1.69 × 10^3^ PEDV genomic copies/mL) and at 28 dpi (2.37 × 10^3^ PEDV genomic copies/mL) for boar F2 (Figure 2). PEDV RNA was detected for longer periods in the sperm-rich fractions than in the seminal fractions. For boar F1, PEDV shedding in sperm-rich fraction was continuous during the first 4 days post-inoculation. Two other shedding phases were observed between 9 and 25 dpi and between 42 and 49 dpi for boar F1.

In the sperm-rich fraction of boar F2, the presence of PEDV RNA was only demonstrated at 2 dpi (2.44 × 10^3^ PEDV genomic copies/mL). The presence of PEDV RNA in this fraction was detected daily until 4 dpi becoming transient with two other phases of excretion observed similar to boar F1 (between 9 and 14 dpi, and 25 and 28 dpi).

The swabs of the prepuce were negative for PEDV RNA at the same time PEDV RNA was detected in semen. PEDV RNA was also detected in the gelatin plug at 18 dpi (9.24 × 10^3^ genomic copies/mL for boar F1 and 4.54 × 10^4^ genome copies/mL for boar F2), at 42 dpi (4.34 × 10^4^ genome copies/mL for boar F1 and 1.58 × 10^5^ genome copies/mL for boar F2) and at 49 dpi for the boar F1 (1.47 × 10^4^ genomic copies/mL).

### PEDV detection in organs

Following necropsy, in all the analyzed organs, PEDV RNA was detected only in the jejunum Peyer’s patches (2.85 × 10^7^ genomic copies/g for boar F1, 1.07 × 10^7^ genomic copies/g for boar F2), the ileum Peyer’s patches (3.88 × 10^6^ genomic copies/g for boar F1, 8.70 × 10^5^ genomic copies/g for boar F2) and the mesenteric lymph nodes (2.41 × 10^5^ genomic copies/g for boar F1, 5.72 × 10^4^ genomic copies/g for boar F2) (Table 2). PEDV RNA was also detected in the ileum of boar F1 (1.65 × 10^6^). No PEDV RNA was detected in the sexual organs (Table 2). No lesions were observed in the gastrointestinal or reproductive tract.

**Table 2:** **PEDV genome loads (number of genome copies/g of tissue) detected in the tissues collected from SPF boars following necropsy**

Boar	Status	Jejunum Peyer’s patches	Ileum Peyer’s patches	Mesenteric nodes	Ileum	Duodenum	Jejunum	Colon	Inguinal nodes	Spleen	Liver	Lung	Psoas muscle	Epididymis					
														Right			Left		
F1	PEDV inoculated	2.85 × 10^7^	3.88 × 10^6^	2.41 × 10^5^	1.65 × 10^6^	0	0	0	0	0	0	0	0	0	0	0	0	0	0
F2	PEDV inoculated	1.07 × 10^7^	8.70 × 10^5^	5.72 × 10^4^	0	0	0	0	0	0	0	0	0	0	0	0	0	0	0

Boar	Testicle						Seminal vesicle				Prostate		Cowper’s glands		Vas deferens		Spermatic cord	
	Right			Left			Right		Left		Right	Left	Right	Left	Right	Left	Right	Left
	Distal pole	Central axis	Apical pole	Distal pole	Central axis	Apical pole	Distal pole	Apical pole	Distal pole	Apical pole								
F1	0	0	0	0	0	0	0	0	0	0	0	0	0	0	0	0	0	0
F2	0	0	0	0	0	0	0	0	0	0	0	0	0	0	0	0	0	0

## Discussion

The clinical signs observed in boars infected with a non-InDel US strain of PEDV in this study were more pronounced than expected. So far, it has been reported that adults, such as boars and sows, show less severe clinical signs than new-born and weaned piglets [5]. In the study reported here, boars demonstrated similar clinical signs (diarrhea, vomiting, and anorexia) compared to what has been described previously in PEDV-infected post-weaning pigs [26]. The duration of virus shedding in boars’ feces determined in our study (16 and 19 days) was also close to values reported for weaned piglets infected with a non-InDel strain, i.e. 17 days [27]. Based on our data from this report, we conclude that boars, irrespective of age, can also show similar clinical signs as young piglets in response to infection with a non-InDel PEDV strain that should not be considered systematically as subclinical in this age.

To the best of our knowledge, the presence of PEDV RNA in semen has never been evaluated previously in experimentally inoculated boars, although sows infected by PEDV may demonstrate reproductive disorders [28]. If sows can be infected by contaminated semen, these data suggested that the trade or dispersion of contaminated semen may lead to a risk of spreading the disease to breeding/gestation and farrowing farms [21, 28]. Several publications emphasized the importance of monitoring semen because of to the large increase in the use of artificial insemination throughout the world. In fact, 90% of sows in Europe, North America and Latin American countries as well as 70% of sows in Thailand and in Taiwan are bred through artificial insemination [21].

Here, we showed the presence of PEDV RNA in the semen of infected specific pathogen-free boars inoculated with a non-InDel strain of PEDV as early as 0.5 dpi. The swabs of the prepuce were negative for PEDV RNA at the same time PEDV nucleic acid was detected in semen. Therefore, it is unlikely that the semen was cross-contaminated with PEDV infected feces during collection of the boars. PEDV RNA was present in both fractions of semen although larger amounts of PEDV were more consistently detected in the sperm-rich fraction which contains sperm and non-sperm cells (spermatozoa, leucocytes, immature germ cells, etc.). In these sperm-rich fractions, the maximum PEDV genomic load reached 3.40 × 10^4^ genomic copies/mL for one boar and 1.75 × 10^4^ genomic copies/mL for the second one. Interestingly, viral excretion in the seminal fractions was transient. Viral shedding in the sperm-rich fraction could be due to viremia or due to irrigation of the genital tract by infected blood cells. Viral shedding in this fraction has already been reported for other porcine viruses such as PCV2 and PRRSV, the latter belonging to the same order, *Nidovirales*, as PEDV [29–31]. For PRRSV, there is currently no consensus among scientists regarding the mechanism of viral shedding in this fraction. One hypothesis is that the virus could be transported by non-sperm cells, such as mononuclear cells or granulocytes. Infected monocytes or macrophages could disseminate the virus through the bloodstream or via the lymph fluid in the genital tract and to semen, without viral replication in the reproductive tissues [30–32]. Another hypothesis for the presence of PRRSV in the sperm-rich fraction is that the virus is directly associated with sperm cells. A previous study showed that PRRSV can infect spermatogenic epithelium and can also infect testicular germ cells of pigs and replicate in these cells. In addition, an association of this virus with macrophages was demonstrated, which is consistent with the previous hypothesis [33]. Both hypotheses mentioned above can be put forward to explain the PEDV shedding in the sperm-rich fraction of infected boars demonstrated in our study. The virus may be associated with non-sperm cells and migrate in the genital tract and in semen, or may be associated with actual sperm cells. Regarding the results of our study, the second hypothesis seems less likely. Following necropsy, no PEDV RNA was found in genital tract tissues. However, RNA degradation during necropsy could not be excluded and would explain a lack of PEDV detection in these tissues. Unfortunately, semen samples were not collected the day of necropsy. These samples would possibly have been negative, consistent with the absence of PEDV RNA detected in sexual organs. We can also hypothesize that infection of genital tract tissues may depend on individual susceptibility with a high degree of variability between animals, and that the number of animals infected with PEDV in our study was too low (2) to consistently detect PEDV RNA in genital tract tissues. As a comparison, in the case of the PRRSV, the virus was found in reproductive organs for few infected boars: in the head of the epididymis (37.5% of boars), in the body of the epididymis (20% of boars), in the tail of the epididymis (12.5% of boars), in the testicles (2.5% of boars), in Cowper’s glands (7.5% of boars), in the seminal vesicles (5% of boars), and in the prostate for 12.5% of boars [34]. The individual variability observed when detecting PRRSV in the reproductive tract is important for the virus pathogenesis and transmission suggesting that PEDV variable detection in reproductive tissues could explain the absence of detection in the two inoculated boars. On necropsy, PEDV RNA was only detected in lymphoid organs including the mesenteric lymph nodes and both jejunum and ileum Peyer’s patches. The presence of extraneous RNA from other viruses of the *Nidovirales* order was already highlighted in lymphoid organs. For example, PRRSV RNA was recovered in lymph nodes from 74.3% of infected boars [34]. Lymphoid tissues contain a large panel of cell types such as innate immune response cells (dendritic cells, macrophages, monocytes or granulocytes) and cells of the adaptive immune response (B and T lymphocytes, primary and secondary lymphoid cells, etc.). We may postulate that PEDV might be associated with one of these cell types, similar to what has been described before for PRRSV and might migrate with these cells to the male genital tract. We can also hypothesize that PEDV replication could be possible in one of these cell types such as dendritic cells (DCs) for example. Interestingly, it has been demonstrated previously that viral replication in DCs is possible for other porcine viruses, including CSFV and PRRSV [35, 36]. This includes the ability of the viruses to use these cells with high migratory capacity to be transported to various sites of the host body. Further studies should be performed to evaluate whether PEDV could use DCs or other cells of lymphoid tissues for replication and/or transportation to the boar genital tract.

The quantity of PEDV genomic copies per milliliter of sperm-rich fraction described in our study was lower than the quantity of genomic copies per milliliter of sperm-rich fraction reported for other porcine viruses. The quantity of PEDV RNA in semen was 10–100 times lower than the quantity found in semen for PCV2 for example. The quantity of recovered PCV2 did not enable infection of the sow by artificial insemination [29]. As result, it could be debated whether artificial insemination could infect a sow considering the PEDV viral titers that were detected in boar semen in this experiment.

We also noted that shedding of PEDV in semen was intermittent. We can make two hypotheses in this regard: the first is that viral shedding is truly intermittent, and the second is that viral shedding in semen is continuous but that the quantity of PEDV RNA is sometimes too low to be detected by RT-qPCR. Intermittent shedding of porcine viruses has already been shown for PCV2, CSF and PRRSV [29, 37–40]. We also observed that shedding of PEDV in semen appeared before shedding in feces and prior to the onset of the clinical signs. This is consistent with the data reported for other porcine viruses, such as CSFV and Aujeszky’s disease virus, which showed viral excretion occurring in semen before the development of clinical signs [21]. Therefore, boars infected by PEDV may be detected after delivery of potentially infected semen. Consequently, specific control measures should be implemented for breeding pigs before semen or boars are imported. This may include the control of seroconversion and control of the absence of previous virus shedding in feces and reporting clinical signs. Implementing these types of measures is particularly important because we have demonstrated that PEDV RNA can be detected in semen even when the boars no longer shed virus in feces and do not show any clinical signs. It would also be beneficial to have more data regarding the prevalence of PEDV among boars used in boar studs.

To conclude, we have demonstrated the shedding of a non-InDel US PEDV strain in the semen (sperm-rich fraction and seminal fraction) of inoculated boars. Further studies are required to determine if the PEDV detected by RT-qPCR in infected semen is potentially infectious. It could be possible to challenge weaned piglets with contaminated semen and observe whether these pigs shed PEDV. If the semen is infectious, it would be necessary to assess whether it can be transmitted by artificial insemination to sows, and evaluate any reproductive disorders.

## Abbreviations

ASFV: African swine fever virus
CSFV: classical swine fever virus
dpi: days post-inoculation
FMDV: foot and mouth disease virus
JBEV: Japanese B encephalitis virus
NGS: next-generation sequencing
PBS: phosphate buffer saline
PCR: polymerase chain reaction
PCV2: porcine circovirus type 2
PEDV: porcine epidemic diarrhea virus
PED: porcine epidemic diarrhea
PPV: porcine parvovirus
PRRSV: porcine reproductive and respiratory syndrome virus
RNA: nucleic acid
RT-qPCR: real-time reverse transcriptase PCR
S/P: sample-to-positive
US: United States
